# The primary lesion apparent diffusion coefficient is a prognostic factor for locoregionally advanced nasopharyngeal carcinoma: a retrospective study

**DOI:** 10.1186/s12885-019-5684-3

**Published:** 2019-05-17

**Authors:** Tao-xiang Huang, Nian Lu, Shan-shan Lian, Hui Li, Shao-han Yin, Zhi-jun Geng, Chuan-miao Xie

**Affiliations:** 1 Department of Radiology, Sun Yat-sen University Cancer Center; State Key Laboratory of Oncology in Southern China, No. 651 Dongfeng Road East, 510060, Guangzhou, People’s Republic of China; 2 Department of Radiology, the Third Affiliated Hospital, Sun Yat-sen University (SYSU), No 600, Tianhe Road, Guangzhou, Guangdong 510630, People’s Republic of China

**Keywords:** Locoregionally advanced nasopharyngeal carcinoma, Magnetic resonance imaging, Apparent diffusion coefficient, Prognosis

## Abstract

**Background:**

To explore prognostic value of the pre-treatment primary lesion apparent diffusion coefficient (ADC) in locoregionally advanced nasopharyngeal carcinoma (LA-NPC).

**Methods:**

A total of 843 patients with newly diagnosed LA-NPC were enrolled from January 2011 to April 2014 and divided into two groups based on ADC values: the low-ADC group and high-ADC group. The 3-year local relapse-free survival (LRFS), distant metastasis free survival (DMFS), disease-free survival (DFS) and overall survival (OS) rates between two groups were compared using Kaplan-Meier curve, and Cox regression analyses were performed to test prognostic value of the pretreatment ADC in LA-NPC.

**Results:**

The cut-off value of the pretreatment ADC for predicting local relapse was 784.5 *×* 10^*−* 6^ mm^2^/s (AUC [area under curve] = 0.604; sensitivity = 0.640; specificity = 0.574), thus patients were divided into low-ADC (< 784.5 *×* 10^*−* 6^; *n* = 473) group and high-ADC (≥784.5 *×* 10^*−* 6^; *n* = 370) group. The low-ADC group had significantly higher 3-year LRFS rate and DFS rate than the high-ADC group (LRFS: 96.2% vs. 91.4%, *P* = 0.003; DFS: 81.4% vs. 73.0%, *P* = 0.0056). Multivariate analysis showed that the pretreatment ADC is an independent prognostic factor for LRFS (HR, 2.04; 95% CI, 1.13–3.66; *P* = 0.017) and DFS (HR, 1.41; 95% CI, 1.04–1.89; *P* = 0.024).

**Conclusions:**

The pretreatment ADC of the primary lesion is an independent prognostic factor for LRFS and DFS in LA-NPC patients.

**Electronic supplementary material:**

The online version of this article (10.1186/s12885-019-5684-3) contains supplementary material, which is available to authorized users.

## Background

Nasopharyngeal carcinoma (NPC) is endemic in South-Eastern Asia while unusual in western countries, the age-standardized incidence rate was 20–50 per 100,000 males in South China [1, 2]. Due to its high sensitivity to radiotherapy and the combination of chemotherapy, the 5-year overall survival rate in patients with stage I-II NPC was over 90%, however, clinical outcomes of patients with stage III-IVA disease are still unsatisfactory because of distant metastasis and local relapse [3, 4]. Retreatment for local relapse is challenging due to fatal complications and unsatisfactory survival, over 50% of treatment-related injuries following re-irradiation were reported [5]. Thus, it is significant to find the high-risk patients for local failure before treatment so as to take more aggressive therapy and administrate shorter follow-up.

Apparent diffusion coefficient (ADC) is obtained from diffusion weighted magnetic resonance imaging (MRI-DWI) after processing, and it is a functional parameter that mainly reflects the Brownian motion of water molecules. Calculating the ADC value of the tumor can quantitatively reflect its intrinsic biological features [6]. To date, the pretreatment ADC value of tumor lesion was reported to be helpful in differential diagnosis [7, 8], tumor staging [9, 10], predicting treatment response of malignancies [11–15]. With regard to NPC, high pretreatment ADC of the primary lesion has been shown to predict poor outcomes and poor response to radio-sensitivity [16, 17]. However, these studies obtained relatively small sample size, resulting in inconclusive results. Moreover, they did not specially focus on local failure. Given the challenging treatment strategy and poor outcome of local failure, it’s worth evaluating the role of the pretreatment ADC in predicting local failure. Therefore, we did this retrospective study to investigate this issue in order to categorize patients and administrate individualized treatment among locoregionally advanced NPC (LA-NPC) patients.

## Methods

### Study patients

**Fig. 1:**
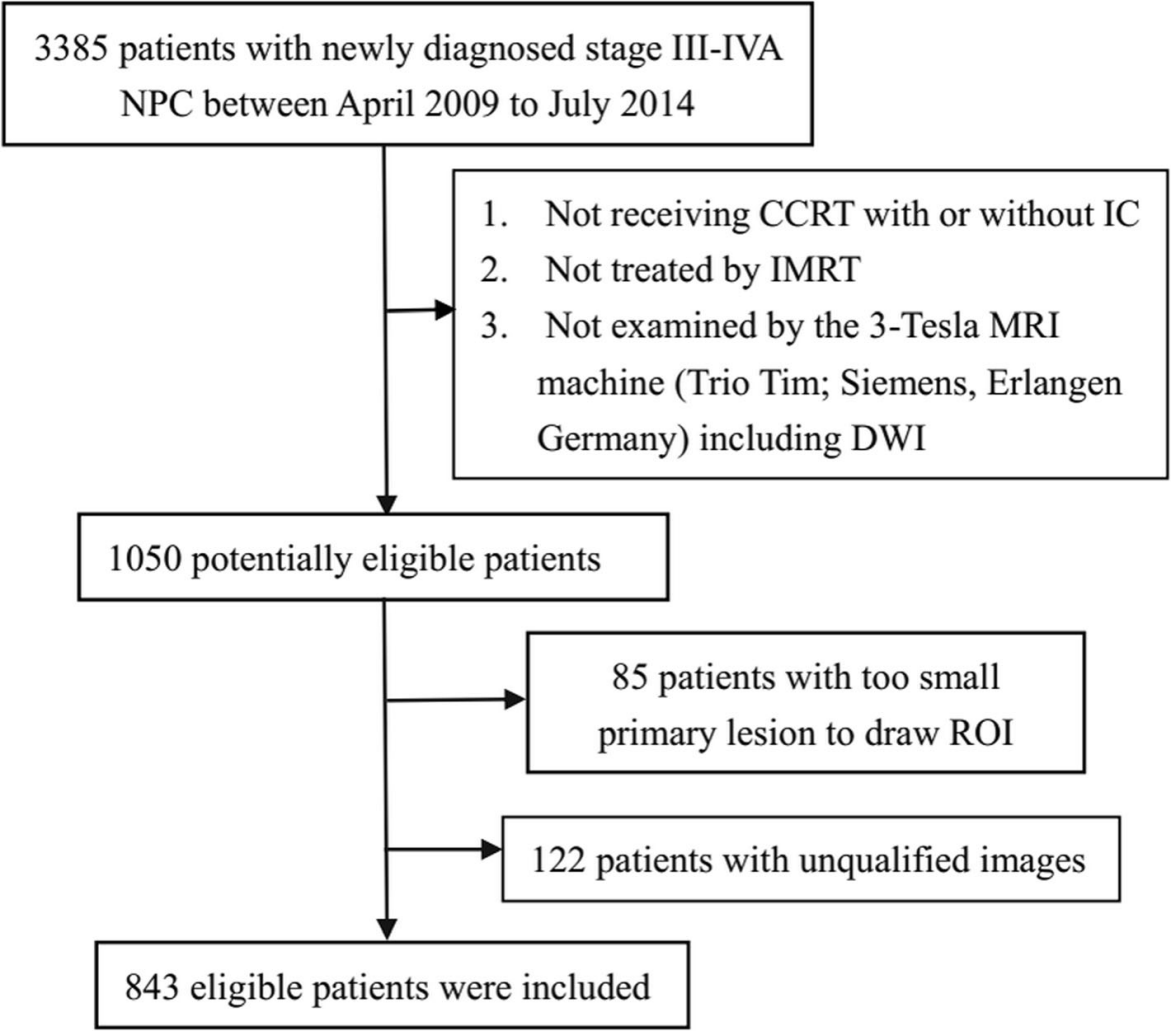
Flowchart of patient inclusion. Abbreviations: NPC = nasopharyngeal carcinoma; CCRT = concurrent chemoradiotherapy; IC = induction chemotherapy; IMRT = intensity-modulated radiotherapy; MRI = magnetic resonance imaging; DWI = diffusion-weighted imaging; ROI = region of interest

The Clinical Research Ethics Committee of Sun Yat-sen University Cancer Center approved the research and the written informed consent was waived. Patients were included in this study if they meet the following inclusion criteria: (i) karnofsky performance score (KPS) ≥ 70; (ii) newly diagnosed stage III-IVA (except T3–4 N0) NPC [18]; (iii) receiving intensity-modulated radiotherapy (IMRT); (iv) the interval between MRI examination and the beginning of treatment was less than 28 days; (v) all patients underwent examination by the same MRI machine (Trio Tim; Siemens, Erlangen Germany) including diffusion-weighted images (DWI) (*b* values: 0, 1000 s/ mm2). Accordingly, a total of 1050 patients between April 2009 and July 2014 were included. In the following analysis, 207 patients were excluded because of unqualified images and difficulties in drawing ROI (region of interest), finally 843 patients were enrolled in this study, which were showed specifically in Fig. 1.

### Clinical staging workup

All patients underwent complete evaluations prior to treatment which consisted of medical history, physical examination, blood test, serum biochemical test, nasopharyngeal fiberscope, MRI of nasopharynx and neck, chest X-ray, abdominal sonography and bone scan. A whole-body 18-fluoro-2-deoxy-glucose (18F-FDG) positron emission tomography with computed tomography (PET/CT) may also be delivered for some patients if necessary.

All patients were regrouped by the 8th edition of the International Union against Cancer/American Joint Committee on Cancer (UICC/AJCC) manual. All imaging data were reviewed by two experienced radiologists (LH, Y-SH) employed at our center to mitigate the heterogeneity, and disagreements were solved by consensus.

### Imaging protocol

MRI examination prior to treatment, from the suprasellar cistern to the collarbone using the same 3-Tesla MRI machine (Trio Tim; Siemens, Erlangen Germany), was done for every patient. Before enhancement, the technician obtained the axial, coronal and sagittal T1-weighted images, the axial T2-weighted MR images and diffusion-weighted images. After the injection of contrast agents, T1-weighted images in axial and sagittal plane and T1-weighted fat-suppressed images in coronal planes were obtained sequentially, all parameters were described in detail (Table 1).

**Table 1:** Parameters of DWI, T1-weighted Imaging and T2-weighted Imaging

Parameter	DWI	T1-weighted Imaging	T2-weighted Imaging
Repetition time (msec)	5600	600	6000
Echo time (msec)	93	8.8	89
B Value	0 and 1000	/	/
Field of view (mm2)	240 (220–260)	240 (220–260)	240 (220–260)
Scan matrix	192 × 192	384 × 307	384 × 307
Section thickness (mm)	10	5	5

Abbreviations: *DWI* = diffusion weighted imaging

**Fig. 2:**
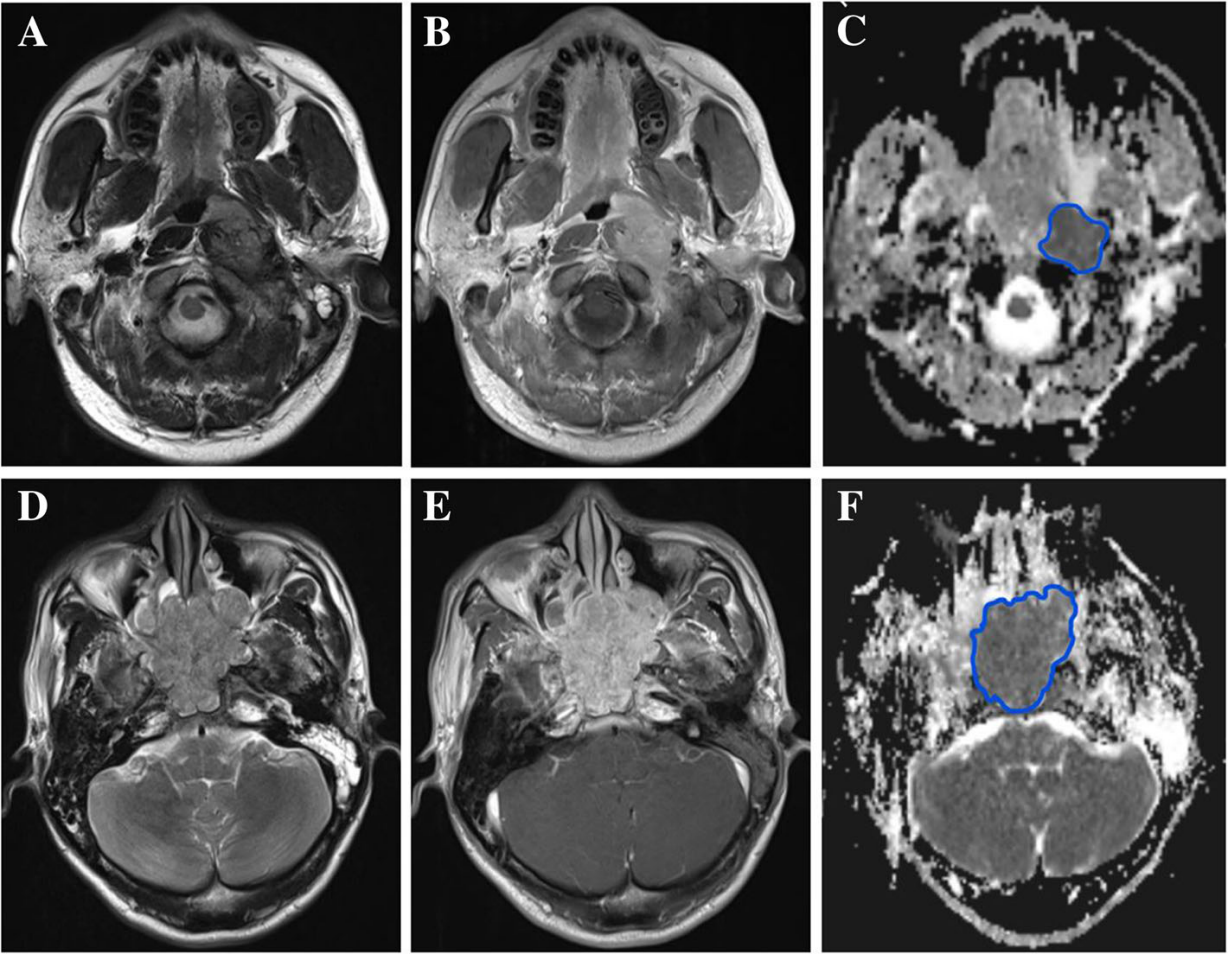
Representative MR images of drawing process:(**a-c**) The axial T2-weighted image, axial enhanced T1 image, axial ADC image of a middle-aged patient, the patient experienced local failure 39.5 months after treatment; (**d-f**:) The axial T2-weighted image, axial enhanced T1 image, axial ADC image of another middle-aged patient, the patient experienced local failure 38.8 months after treatment. Abbreviations: ADC = apparent diffusion coefficient.

The ADC value was quantified by calculating the signal intensity (SI) of each pixel of the tumor area using the following eq. *SI = SI0e-bD*, where SI means the measurement of signal intensity, b means b value, D means ADC, SI0 means SI when *b*-value is 0. Choosing 1000 s/mm2 as a *b*-value to calculate the D value in this study and each patient owned a corresponding ADC map after processing. Mean ADC value of lesion was obtained by drawing the two-dimensional region of interest (ROI) to cover as much as the primary lesion in the slice with largest diameter, discarding the necrotic and cystic part. The consensus criteria of ROI drawing was set first by two experienced radiologists (LH, Y-SH) with more than 10-year experience in NPC imaging diagnosis, and they conducted the drawing process without knowing the clinical outcomes. Figure 2 showed two cases with corresponding ADC maps.

### Treatment and follow up

All of the 843 patients underwent IMRT using the simultaneous integrated boost (SIB) technique. The prescribed doses were 66–72 Gy, 64–70 Gy, 60–63 Gy, 50–56 Gy to the planning target volume (PTV) of nasopharyngeal gross tumor volume (GTV), the PTV of GTV of metastatic lymph nodes, the PTV of high-risk clinical target volume, the PTV of low-risk clinical target volume, respectively. Moreover, 40.6% (343/843) of patients received induction chemotherapy (IC) plus concurrent chemoradiotherapy (CCRT), while 59.3% (59.3%) of patients received CCRT alone. IC consist of cisplatin-based regimens delivered every three weeks for 2–3 cycles. Concurrent chemotherapy was weekly or tri-weekly cisplatin.

During the first two years after treatment, followed-up was conducted every three months, then every 6 months in years 3–5 and thereafter once a year. The standard follow-up includes complete medical history, physical examination and MRI of the neck and nasopharynx, chest CT and abdominal sonography. All local recurrence and distant metastasis was determined by biopsy results or highly probable imaging finding on CT, MRI or PET-CT.

### Statistical analysis

In this study, the first endpoint was local relapse-free survival (LRFS) defined as the time interval between pathology diagnosis and first local recurrence. Other endpoints included distant metastasis-free survival (DMFS, time interval from diagnosis to first distant metastasis), disease-free survival (DFS, time interval from diagnosis to first disease progression) and overall survival (OS, time interval from diagnosis to death due to any cause or patient censoring).

For predicting local relapse, receiver-operating characteristic (ROC) curve were done to determine the cut-off value (the maximal conditional Youden score) of the pretreatment ADC [19]. Based on this value, the patients were divided into low-ADC group and high-ADC group. Categorical or continuous variables (age, gender, drinking, smoking, family history of cancer, serum Epstein-Barr virus load, serum lactate dehydrogenase [LDH], T classification, N classification, clinical stage, treatment methods) between high-risk and low-risk groups were compared using the Chi-square test or non-parametric test.

Survival analysis including LRFS, DMFS, DFS and OS was done using the Kaplan-Meier method and compared by the log-rank test. In order to test the independent prognostic value of the pretreatment ADC, the host factors (age, gender, drinking, smoking, family history of cancer), biochemical results (pretreatment serum EBV load, serum LDH), tumor stage (T classification, N classification, overall stage) and treatment methods (CCRT or IC + CCRT) were included into multivariate analysis using Cox proportional hazards model, and hazard ratio (HR) and 95% confidence interval (CI) were obtained. A two-sided *P* value that less than 0.05 was considered significant. SPSS software (version 21.0; SPSS, Chicago, Ill) was used for all analyses.

## Results

### Patient baseline characteristics

A total of 843 patients were enrolled in this study, the male (*n* = 609) to female (*n* = 234) ratio is 2.60:1. Median age for the whole group was 44 years (range, 9–74 years). Moreover, most (96.7%) of patients were histo-pathologically confirmed undifferentiated non-keratinizing carcinoma. Additionally, 57.7% (487/843) of patients had stage III disease while 42.2% (356/843) had stage IVA disease. In total, 343 (40.6%) patients received IC + CCRT and 500 (59.4%) received CCRT alone (Table 2).

**Table 2:** Baseline characteristics of 843 patients with LA-NPC

	Low-ADC group	High-ADC group	*P*-value ^a^
Total	473	370	
Age(years)			0.732
Median(range)	45(9–74)	44 (12–72)	
< 44	236 (49.9%)	189 (51.1%)	
≥44	237 (50.1%)	181 (48.9%)	
Gender			0.039
Male	355 (75.1%)	254 (68.6%)	
Female	118 (24.9%)	116 (31.4%)	
EBV-DNA			0.960
Median(range)	8.0 (0–5150.0)	6.0 (0–2810.0)	
< 40.0	351 (74.2%)	274 (74.1%)	
≥40.0	122 (25.8%)	96 (25.9%)	
LDH (U/L)			0.189
Median (range)	183.2 (1.7–396.5)	178.8 (108.0–753.0)	
< 245.0	431 (91.1%)	327 (88.4%)	
≥245.0	42 (8.9%)	43 (11.6%)	
WHO histologic type			0.047
KSCC	2 (0.4%)	4 (1.1%)	
DNKC	7 (1.5%)	14 (3.8%)	
UNKC	464 (98.1%)	352 (95.1%)	
Smoking			0.596
No	292 (61.7%)	235 (63.5%)	
Yes	181 (38.3%)	135 (36.5%)	
Drinking			0.593
No	395 (83.5%)	314 (84.9%)	
Yes	78 (16.5%)	56 (15.1%)	
Family history of cancer			0.966
No	356 (75.3%)	278 (75.1%)	
Yes	117 (24.7%)	92 (24.9%)	
T classification ^b^			0.924
T1–2	65 (13.7%)	50 (13.5%)	
T3–4	408 (86.3%)	320 (86.5%)	
N classification ^b^			0.359
N0–1	263 (55.6%)	194 (52.4%)	
N2–3	210 (44.4%)	176 (47.6%)	
Overall stage^b^			0.550
III	269 (56.9%)	218 (58.9%)	
IVA	204 (43.1%)	152 (41.1%)	
Treatment			0.529
IC + CCRT	188 (39.7%)	155 (41.9%)	
CCRT	285 (60.3%)	215 (58.1%)	

Abbreviation: *LA-NPC* locoregionally advanced nasopharyngeal carcinoma, *EBV-DNA* pretreatment serum Epstein-Barr virus load, *ADC* apparent diffusion coefficient, *LDH* lactate dehydrogenase, *IC* induction chemotherapy, *CCRT* concurrent chemoradiotherapy, *KSCK* keratinizing squamous cell carcinoma, *DNKC* differentiated non-keratinizing carcinoma, *UNC* undifferentiated non-keratinizing carcinoma

^a^*P* values were calculated by Chi-square test.

^b^According to the 8th edition of UICC/AJCC staging system

### Cut-off value of ADC

Of all the patients, the median of the pretreatment ADC was 771.0 × 10^− 6^ mm2/s (range, 370.0 to 1051.0× 10^− 6^ mm2/s). ROC analysis identified the cut-off value of the pretreatment ADC for predicting local relapse as 784.5 *×* 10^*−* 6^ mm2/s (maximal Youden score = 0.214; sensitivity = 0.640; specificity = 0.574; AUC [area under the ROC] = 0.604 [95%CI, 0.533–0.676]). Accordingly, patients were divided into low-ADC group (< 784.5 *×* 10^*−* 6^ mm2/s) (*n* = 473) and high-ADC group (≥ 784.5 *×* 10^*−* 6^ mm2/s) (*n* = 370) based on the value. The demographics and clinical characteristics were described in Table 1 and were well balanced.

### Failure patterns

Up to the last visit, the median follow-up duration was 54.9 months (range, 3.3–83.6 months). In total, 18 (3.8%) patients in low-ADC group and 32 (8.6%) patients in high-ADC group experienced local relapse, 64 (13.5%) patients in low-ADC group and 60 (16.2%) patients in high-ADC group suffered distant metastasis. Accordingly, 88 (18.6%) and 100 (27.0%) patients in low-ADC and high-ADC groups developed treatment failure. Consequently, 53 (11.2%) patients in low-ADC and 57 (15.4%) patients in high-ADC group died.

### Survival analysis

**Fig. 3:**
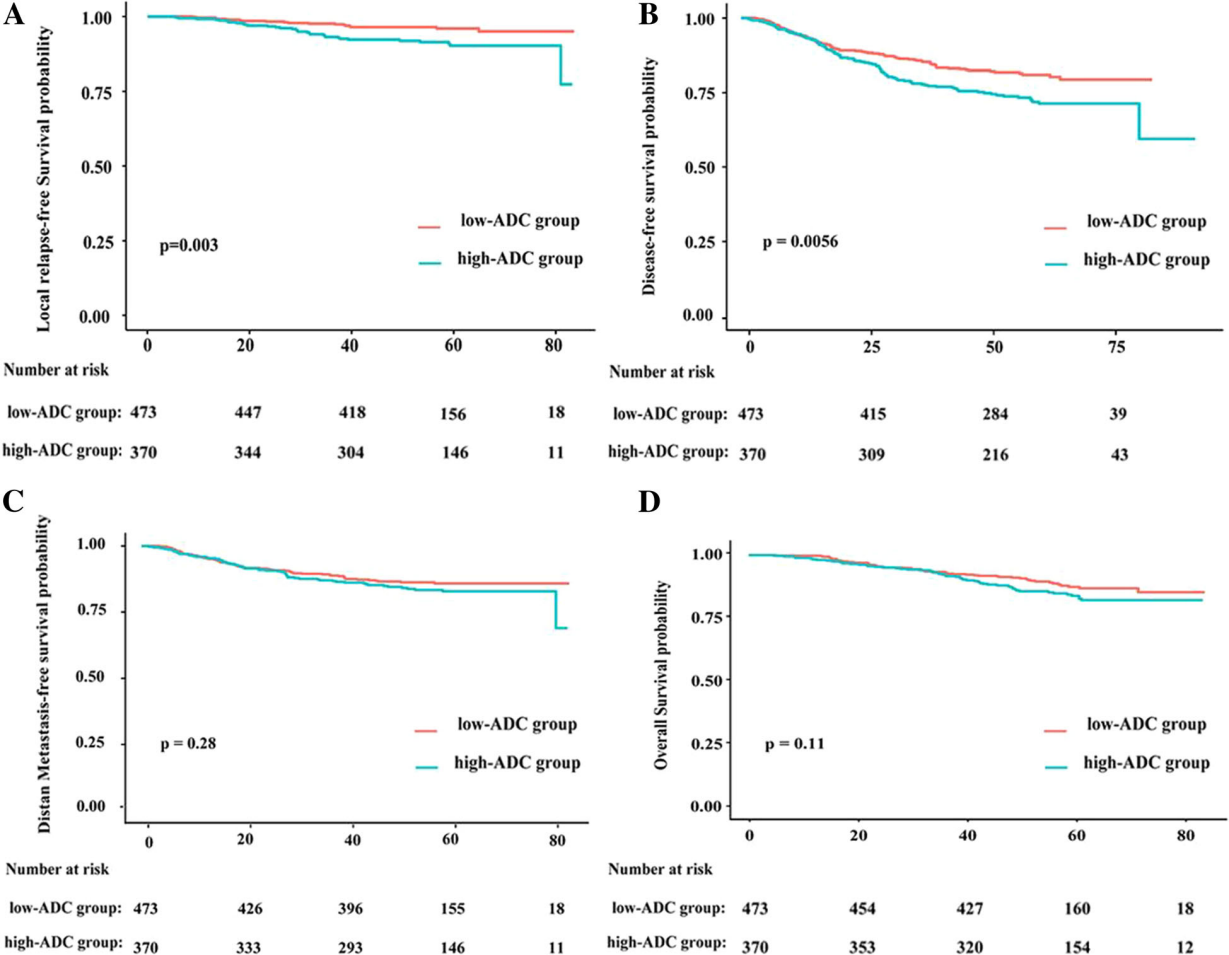
Kaplan-Meier (**a**) local relapse-free survival (LRFS), (**b**) disease-free survival (DFS), (**c**) distant metastasis-free survival (DMFS), (**d**) overall survival (OS) curves for the selected 843 patients with stage III-IVA nasopharyngeal carcinoma (except T3–4 N0) between low-ADC group and high-ADC group; low-ADC group = patients with a primary lesion ADC value prior to treatment < 0.784.5 × 10–3 mm2/s; high-ADC group = patients with a primary lesion ADC value prior to treatment ≥0.784.5 × 10–3 mm2/s

The low-ADC group had significantly higher 3-year LRFS rate and DFS rate than the high-ADC group (LRFS: 96.2% vs.91.4%, *P* = 0.003; DFS: 81.4% vs. 73.0%, *P* = 0.0056). while 3-year DMFS rate and OS rate were found no significant difference between groups (DMFS: 86.5% vs. 83.8%, *P* = 0.28; OS: 88.8% vs. 84.6% *P* = 0.11) (Figure 3). After adjusting for various factors, multivariate analysis showed that the pretreatment ADC is an independent prognostic factor for LRFS (HR, 2.04; 95% CI, 1.13–3.66; *P* = 0.017) and DFS (HR, 1.41; 95% CI, 1.04–1.89; *P* = 0.024) (Table 3). In addition , Propensity score matching identified 356 well-matched pairs, survival analysis showed consistent results (Additional file 1: Figure S1 and Additional file 2: Table S1).

**Table 3:** Multivariate analysis of prognostic factors in the 843 patients with LA-NPC

Endpoints	Variable	HR (95% CI)	*P* value ^a^
LRFS	T classification ^b^	5.919 (1.386–25.27)	0.016
	N classification ^b^	1.836 (1.028–3.280)	0.040
	Mean ADC	2.039 (1.135–3.664)	0.017
	Treatment	0.551 (0.307–0.989)	0.046
OS	N classification ^b^	1.689 (1.144–2.493)	0.008
	LDH	2.204 (1.364–3.560)	0.001
DFS	N classification ^b^	1.626 (1.161–2.278)	0.004
	LDH	2.13 (1.447–3.146)	< 0.001
	Mean ADC	1.409 (1.045–1.899)	0.024
DMFS	EBV-DNA	1.701 (1.587–2.161)	0.006
	Gender (Male)	0.519 (0.324–0.830)	0.006
	LDH	2.624 (1.709–4.029)	< 0.001

Abbreviations: *Mean ADC* mean value of the primary lesion apparent diffusion coefficient, *EBV-DNA* pretreatment serum Epstein-Barr virus load, *CI* confidence interval, *HR* hazard ratio, *LA-NPC* locoregionally advanced nasopharyngeal carcinoma, *LRFS* Local relapse-free survival, *OS* Overall survival, *DFS* Disease-free survival, *DMFS* Distant metastasis-free survival

^a^*P* values were calculated using an adjusted Cox proportional hazards model including the following factors: the host factors (age, gender, drinking, smoking, family history of cancer), biochemical results (pretreatment serum EBV load, serum LDH), tumor stage (T category, N category, overall stage) and treatment method (CCRT or IC + CCRT)

^b^According to the 8th edition of UICC/AJCC staging syst

## Discussion

Our Current study identified the pretreatment ADC of the primary lesion as an independent prognostic factor for patients with LA-NPC that patients with high-ADC achieved significantly worse 3-year LRFS rate and DFS rate compared with patients with low-ADC, indicating that the pretreatment ADC could act as a useful prognostication for future management of LA-NPC.

Nowadays, treatment strategy for NPC is decided mainly based on clinical TNM stage, which only takes the anatomical change into consideration. Based on this standard reference, the 5-year OS rates were 60–85% for patients with stage III–IVA disease and far from satisfaction [3, 20]. Therefore, it is essential to find out the subgroup of patients who achieved poor treatment response. MR-DWI is an non-invasive assessment of the intrinsic biological features of tissues including microvasculature, cell density, membrane permeability and so on [6]. In many malignancies, the ADC has been proved to be valuable for differential diagnosis, prediction of lymph node metastasis and prognostic evaluation [21–25]. Several studies have reported that high ADC value before treatment correlated with poor treatment responses and survival outcomes in brain tumor, bladder cancer and head and neck cancers [26, 27]. On the contrary, another research studied patients with renal cancer and found that low ADC value predicted higher rate of metastasis after surgery [28], suggesting that ADC can serve as a useful pre-treatment indicator for many malignancies. Similarly, our study also validate that pre-treatment ADC could serve as an important prognostication in NPC.

The underlying mechanism for our finding may be that high pre-treatment ADC level usually predicts more invasive biological features of tumor. Tumor lesions with high ADC may have more invisible micronecrosis and inflammation, suggesting that hypoxia should exist [29] and therefore results in lower radio-sensitivity [30, 31]. Moreover, more micronecrosis and hypoxia indicated that tumor is inclined to grow fast, leading to higher invasive potential of malignant cells and higher local relapse [32, 33]. With regard to our findings that DMFS did not significantly differ between the high-ADC and low-ADC groups, the major reason may be that distant metastasis had a stronger association with lymph node metastasis than local lesion relapse [4].

Several previous researches regarding to NPC reported controversial conclusions. Razek et al. studied 30 patients with NPC and found that low pre-treatment ADC was associated with larger tumor size and higher rate of lymph node metastasis; however, all patients enrolled in that study were from non-endemic area and the pathological type distribution is different from that in our research [34]. Chen et al. recruited 31 patients with stage III-IV disease and reported that patients with high pretreatment ADC values and large ADC increase early after neoadjuvant chemotherapy were likely to respond better to chemoradiotherapy. Notably, both of Chen’s and Razek’s studies had small sample size, and didn’t investigated the relationship between pretreatment ADC and long-term survival [35]. Consistent with our findings, Zhang et al. also found that high pretreatment ADC predict higher local failure rate [16]. Compared with that study, our research included more patients (834 vs. 541) and specially focus on local relapse. Moreover, we only focus on the LA-NPC rather than all stage patients. Thus our research had a larger sample size and particularly focus on the LA-NPC subgroup that has higher rate of local relapse. Finally, we included biochemical and treatment factors into multivariate analysis, making our results more conclusive.

However, there were still some limitations in our study. First, it was a retrospective study. Although we had set up strict inclusion criteria and applied multivariate analysis to establish the significant factors, potential bias related to the nature of retrospective study could not be avoided totally. Second, there were no standard scanning parameters for NPC patients. Different types of MR machine and different b values selection will influence the calculation of the pretreatment ADC. Third, it was difficult to draw region of interest (ROI) for small lesions, and consensus had not met yet whether to draw several small circles of ROI or draw a big ROI covering the whole lesion. Furthermore, the AUC of pretreatment ADC was 0.604, which was not enough to be used in treatment strategy decision [19].

## Conclusion

In summary, the pretreatment ADC is an independent prognostic factor for LRFS and DFS in LA-NPC patients, which might be used to select patients at high risk of local failure and therefore administrate intense treatment. More researches combining the pretreatment ADC, TNM stage and other factors or using radiomics or convolutional neural network (CNN) based on ADC images are warranted to be done to increase predictive or prognostic efficacy in the future.

## Additional files

Additional file 1: **Figure S1.** Kaplan-Meier (A) local relapse-free survival (LRFS), (B) disease-free survival (DFS), (C) distant metastasis-free survival (DMFS), (D) overall survival (OS) curves for the 356 pairs identified by propensity score matching; low-ADC group= patients with a primary lesion ADC value prior to treatment < 0.784.5 × 10−3 mm2/s (*n*=356); high-ADC group = patients with a primary lesion ADC value prior to treatment ≥ 0.784.5 × 10−3 mm2/s (*n*=356). (TIF 2663 kb)

Additional file 2: **Table S1.** Baseline characteristics of propensity score matched 356 pairs patients with LA-NPC. Abbreviations: Mean ADC = mean value of the primary lesion apparent diffusion coefficient; EBV-DNA = pretreatment serum Epstein-Barr virus load; CI = confidence interval; HR = hazard ratio; LA-NPC = locoregionally advanced nasopharyngeal carcinoma; LRFS = Local relapse-free survival; OS = Overall survival; DFS = Disease-free survival; DMFS = Distant metastasis-free survival. a *P* values were calculated using an adjusted Cox proportional hazards model including the following factors: the host factors (age, gender, drinking, smoking, family history of cancer), biochemical results (pretreatment serum EBV load, serum LDH), tumor stage (T category, N category, overall stage) and treatment method (CCRT or IC+CCRT). b According to the 8th edition of UICC/AJCC staging system. (DOCX 20 kb)

## Abbreviations

ADC: Apparent diffusion coefficient
AUC: Area under curve
CCRT: Concurrent chemoradiotherapy
CIs: Confidence intervals
CT: Computed tomography
CTV: Clinical target volume
DFS: Disease-free survival
DMFS: Distant metastasis-free survival
DWI: Diffusion-weighted imaging
EBV: Epstein-Barr virus
GTV: Gross tumour volume
HR: Hazard ratio
IC: Induction chemotherapy
IMRT: Intensity-modulated radiotherapy
LA-NPC: Locoregionally advanced nasopharyngeal carcinoma
LDH: Lactate dehydrogenase
LRFS: Local relapse-free survival
MRI: Magnetic resonance imaging
NCCN: National comprehensive cancer network
OS: Overall survival
PET: Positron emission tomography
PTV: Planning target volume
ROC: Receiver operating characteristic
UICC/AJCC: International Union against Cancer/American Joint Committee on Cancer
