# Supplementary figures and images for: The primary lesion apparent diffusion coefficient is a prognostic factor for locoregionally advanced nasopharyngeal carcinoma: a retrospective study
Source: BMC Cancer. 2019 May 17;19:470. doi: 10.1186/s12885-019-5684-3 (PMC6525458; doi:10.1186/s12885-019-5684-3)

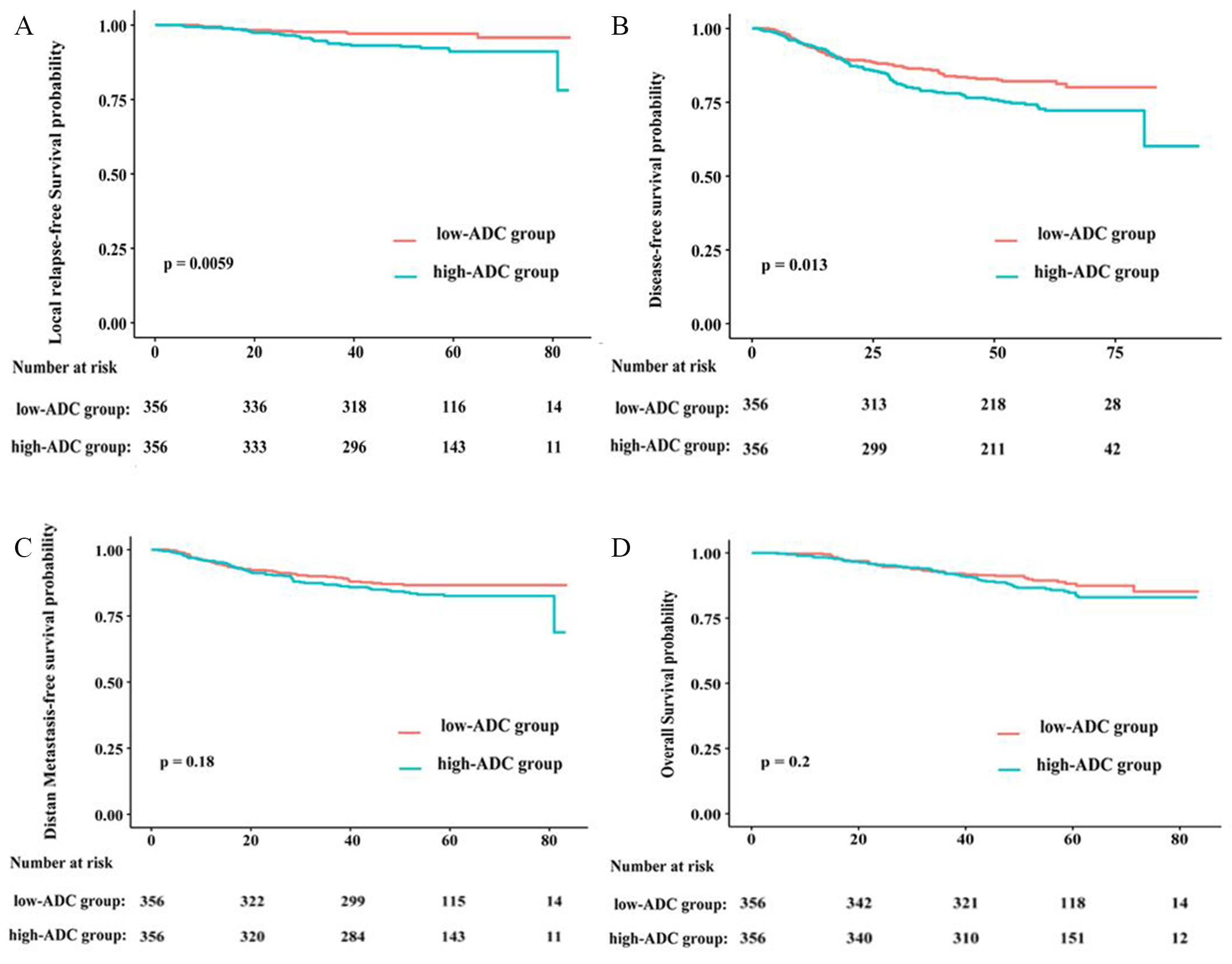

Supplement: Supplementary file 1 — Figure S1. Kaplan-Meier (A) local relapse-free survival (LRFS), (B) disease-free survival (DFS), (C) distant metastasis-free survival (DMFS), (D) overall survival (OS) curves for the 356 pairs identified by propensity score matching; low-ADC group= patients with a primary lesion ADC value prior to treatment < 0.784.5 × 10−3 mm2/s (n=356); high-ADC group = patients with a primary lesion ADC value prior to treatment ≥ 0.784.5 × 10−3 mm2/s (n=356). (TIF 2663 kb) [file 12885_2019_5684_MOESM1_ESM.tif]
