# Supplementary material for: The primary lesion apparent diffusion coefficient is a prognostic factor for locoregionally advanced nasopharyngeal carcinoma: a retrospective study
Source: BMC Cancer. 2019 May 17;19:470. doi: 10.1186/s12885-019-5684-3 (PMC6525458; doi:10.1186/s12885-019-5684-3)
Supplement: Supplementary file 2 — Table S1. Baseline characteristics of propensity score matched 356 pairs patients with LA-NPC. Abbreviations: Mean ADC = mean value of the primary lesion apparent diffusion coefficient; EBV-DNA = pretreatment serum Epstein-Barr virus load; CI = confidence interval; HR = hazard ratio; LA-NPC = locoregionally advanced nasopharyngeal carcinoma; LRFS = Local relapse-free survival; OS = Overall survival; DFS = Disease-free survival; DMFS = Distant metastasis-free survival. a P values were calculated using an adjusted Cox proportional hazards model including the following factors: the host factors (age, gender, drinking, smoking, family history of cancer), biochemical results (pretreatment serum EBV load, serum LDH), tumor stage (T category, N category, overall stage) and treatment method (CCRT or IC+CCRT). b According to the 8th edition of UICC/AJCC staging system. (DOCX 20 kb) [file 12885_2019_5684_MOESM2_ESM.docx]

**Supplementary table1.** Baseline characteristics of propensity score matched 356 pairs patients with LA-NPC.

|  | Low-ADC group | High-ADC group | *P*-value ^a^ |
| --- | --- | --- | --- |
| Total | 356 | 356 |  |
| Age (years) |  |  | 0.987 |
| Median (range) | 45 (10-74) | 44 (12-72) |  |
| Gender |  |  | 0.808 |
| Male | 245 (68.8%) | 248 (69.7%) |  |
| Female | 111 (31.2%) | 108 (30.3%) |  |
| EBV-DNA |  |  | 1.000 |
| Median (range) | 7.3 (0-5150.0) | 5.9 (0-2740.0) |  |
| <40.0 | 264 (74.1%) | 264 (74.1%) |  |
| ≥40.0 | 92 (25.9%) | 92 (25.9%) |  |
| LDH (U/L) |  |  | 0.635 |
| Median (range) | 184 (1.7-396.5) | 178.0 (108.0-753.0) |  |
| <245.0 | 318 (89.3%) | 314 (88.2%) |  |
| ≥245.0 | 38 (10.7%) | 42 (11.8%) |  |
| WHO* |  |  | 0.772 |
| DNKC | 5 (1.4%) | 7 (1.97%) |  |
| UNKC | 351 (98.6%) | 349 (98.0%) |  |
| Smoking |  |  | 0.817 |
| No | 220 (61.8%) | 223 (62.6%) |  |
| Yes | 136 (38.2%) | 133 (37.4%) |  |
| Drinking |  |  | 1.000 |
| No | 301 (83.5%) | 301 (83.5%) |  |
| Yes | 55 (16.5%) | 55 (16.5%) |  |
| Family history of cancer | |  | 0.863 |
| No | 264 (75.0%) | 267 (75.4%) |  |
| Yes | 92 (25.0%） | 89 (24.7%) |  |
| T classification ^b^ |  |  | 0.591 |
| T1-2 | 53 (14.9%) | 48 (13.5%) |  |
| T3-4 | 303 (85.1%) | 308 (86.5%) |  |
| N classification ^b^ |  |  | 0.599 |
| N0-1 | 193 (54.2%) | 186 (52.3%) |  |
| N2-3 | 163 (45.8%) | 170 (47.7%) |  |
| Overall stage ^b^ |  |  | 1.000 |
| III | 209 (58.7%) | 209 (58.7%) |  |
| IVA | 147 (41.3%) | 147 (41.3%) |  |
| Treatment |  |  | 0.762 |
| IC+CCRT | 154 (43.3%) | 150 (42.1%) |  |
| CCRT | 202 (56.7%) | 206 (57.9%) |  |

Abbreviation: LA-NPC = locoregionally advanced nasopharyngeal carcinoma; EBV-DNA = pretreatment serum Epstein-Barr virus load; ADC = apparent diﬀusion coefficient; LDH = lactate dehydrogenase; IC = induction chemotherapy; CCRT = concurrent chemoradiotherapy; KSCK = keratinizing squamous cell carcinoma; DNKC = differentiated non-keratinizing carcinoma; UNC = undifferentiated non-keratinizing carcinoma.

^a^ *P* values were calculated by Chi-square test.
^b^ According to the 8th edition of UICC/AJCC staging system.
